# Inhaled fluticasone propionate impairs pulmonary clearance of *Klebsiella Pneumoniae* in mice

**DOI:** 10.1186/1465-9921-13-40

**Published:** 2012-05-31

**Authors:** Craig M Patterson, Richard L Morrison, Alain D’Souza, Xu S Teng, Kyle I Happel

**Affiliations:** 1 Department of Medicine, Section of Pulmonary and Critical Care Medicine, Louisiana State University Health Sciences Center, New Orleans, LA, USA; 2 Department of Physiology, Louisiana State University Health Sciences Center, New Orleans, LA, USA; 3 Department of Medicine, Section of Pulmonary and Critical Care Medicine, Louisiana State University Health Sciences Center, 1901 Perdido St., Suite 3205, New Orleans, LA, 70112, USA

## Abstract

**Background:**

Recent trials demonstrate increased pneumonia risk in chronic obstructive pulmonary disease patients treated with the inhaled corticosteroid (ICS) fluticasone propionate (FP). There is limited work describing FP effects on host defenses against bacterial pneumonia.

**Methods:**

C57BL/6 mice received daily, nose-only exposure to nebulized FP or vehicle for 8 days, followed by pulmonary challenge with *Klebsiella pneumoniae*. Bacterial burden, phagocytosis, leukocyte recruitment, cytokine expression, nitric oxide release, and survival were measured.

**Results:**

Inhaled FP increased bacterial burden in lungs and blood 48 h after infection but affected neither in vivo phagocytosis of bacteria by alveolar macrophages (AM) nor alveolar neutrophil recruitment. AM from FP-treated mice showed impaired expression of infection induced TNF-alpha, IP-10 (CXCL-10), and interleukin 6 (IL-6), and AM also showed a trend towards impaired intracellular pathogen control following in vivo infection. In vitro FP treatment resulted in a dose-dependent impairment of cytokine expression by AM. Furthermore, infection-induced nitric oxide (but not hydrogen peroxide) production was impaired by FP in vivo and in vitro. FP decreased survival in this model.

**Conclusions:**

Exposure to inhaled FP impairs pulmonary clearance of *K. pneumoniae* in mice, an effect associated with greater systemic bacteremia and death. Decreased AM cytokine and nitric oxide expression parallel the failure to control infection. These results support the study of ICS effects on human pulmonary host defenses.

## Introduction

The immune modulatory effects of inhaled corticosteroids (ICS) on inflammatory airway diseases are well-recognized, and they are widely used in the treatment of asthma [1]. In recent years, ICS have also been increasingly utilized in the treatment of chronic obstructive pulmonary disease (COPD) and other pulmonary disease states [2]. Several large, randomized trials have demonstrated significant clinical benefits of ICS, such as fewer acute COPD exacerbations, better lung function, and improved symptom scores [3-5]. Despite these benefits, multiple trials have now revealed an increased incidence of pneumonia diagnosis in COPD patients given ICS, particularly fluticasone propionate [6-9]. Despite the large numbers of patients in these trials, heterogeneity in data collection and the lack of a prospective definition for pneumonia confound a definitive causal link between ICS use and pneumonia.

The effects of glucocorticoids (both systemic and inhaled) on airways inflammation are well-documented and have led to the widespread use of these medications in the treatment of obstructive lung diseases. A meta-analysis of 18 randomized controlled trials (totaling nearly 17,000 patients) to ascertain the risk of pneumonia associated with ICS use by COPD patients concluded that ICS use is associated with a significantly increased risk of serious pneumonia, without a significantly increased risk of death [10]. A subsequent trial studying a lower dose of FP (250mcg twice daily) in COPD patients also reported increased pneumonia rates in subjects receiving ICS [8]. While strict microbiologic or radiographic criteria were not required for diagnosing pneumonia in these trials, it is intuitive that several aspects of pulmonary host defense may be affected by ICS given their potent anti-inflammatory effects.

We hypothesized that clinically relevant doses of inhaled FP administered via *nose-only* aerosolized exposure alters innate pulmonary defenses to live bacterial challenge with a typical pneumonia pathogen. We chose a murine model of pulmonary infection with *Klebsiella pneumoniae* given our familiarity with this model and a subsequent analysis of the TORCH trial data identifying this organism as an etiologic agent of pneumonia in patients treated with ICS [11].

## Materials and methods

### Mice

Specific pathogen-free male C57BL/6 mice (Charles River Laboratories; Wilmington, MA) between the ages of 6 – 8 weeks were used for all experiments. All mice were housed in the LSUHSC vivarium and treated in accordance with institutional guidelines. Mice received food and water ad libitum during 12-hour light/dark cycles. All procedures were approved by the LSUHSC Institutional Animal Care and Use Committee.

### Fluticasone propionate administration and intratracheal injection

FP powder (Sigma, St. Louis, MO) was resuspended at a concentration of 300 μg/mL in sterile saline with 0.17% *vol/vol* Tween-80. All mice received daily 20 minute exposures to nebulized FP solution (or vehicle control) via a nose-only aerosolized delivery (InExpose system; SCIREQ, Montreal, QC, Canada) for eight successive days. This dosing was modeled after a previously published mouse FP study and is felt to approximate a 1 mg daily dose of FP in humans [12]. After the eighth daily exposure, mice were anesthetized by intraperitoneal injection of ketamine-xylazine solution and underwent intratracheal administration of 1x10^4^ CFU *Klebsiella pneumoniae* (serotype 43816; ATCC, Manassas, VA). *n* ≥ 8 per group for all in vivo experiments.

### Blood, lung, and bronchoalveolar lavage harvest

At designated time points, mice were anesthetized with isofluorane and killed by diaphragmatic interruption. Whole blood was obtained by right ventricular puncture. Lungs were removed en bloc and homogenized in 1 ml of sterile PBS for microbiological studies, or in 1 mL of buffer RLT (RNeasy Mini Kit; Qiagen, Valencia, CA) for mRNA isolation, or in 1 mL of PBS with 0.1% Triton X-100 and a protease-inhibitor cocktail (Complete Mini, Roche Diagnostics; Indianapolis, IN) for protein analysis. Bronchoalveolar lavage (BAL) cells were retrieved by lavage of removed lungs with PBS containing 0.1% glucose. A total of 10 mL lavage was performed in 1 mL aliquots. The initial 1 mL BAL aliquot was used for cytokine analysis.

### Bacteriology

Bacterial CFU in blood and lungs were determined by plating 100 μl of whole blood (and its serial dilutions) or homogenized lung (and its serial dilutions) on MacConkey agar plates (Oxoid; Basingstoke, Hampshire, England) followed by overnight incubation.

### In vivo phagocytosis

Mice were administered 1 × 10^9^ eGFP-*Klebsiella pneumoniae* (serotype 43816, a kind gift from Steven Clegg, University of Iowa) by intratracheal inoculation. After 2 hours, mice were killed and BAL was performed to retrieve alveolar macrophages. Cells were centrifuged at 100 × g and washed 3 times to remove free bacteria. The fluorescence of phagocytosed organisms within alveolar macrophages was then measured by flow cytometry (FACSCalibur™; BD Biosciences, San Jose, CA). The percentage of cells phagocytosing bacteria and the mean fluorescence intensity (MFI) of each cell were recorded. Examination of BAL cells by deconvolution microscopy confirmed that fluorescent organisms were intracellular, as very few fluorescing AM (<1%) showed cell surface-associated bacteria.

### Cell culture

AM from FP and vehicle-nebulized mice were retrieved by BAL as described above. 2x10^5^ cells per well were plated in 96 well plates in DMEM + 10% fetal calf serum. Cells were exposed to *K. pneumoniae* at a multiplicity of infection (MOI) of 100:1. Culture supernatant was harvested for cytokine or reactive oxygen/nitrogen species analysis at indicated time points.

### Reactive oxygen/nitrogen species

In vitro production of hydrogen peroxide by alveolar macrophages in response to 10nM PMA and 1 μM calcium ionophore A23187 (both from Sigma) was assessed using the fluorogenic Amplex® UltraRed reagent (Invitrogen; Eugene, OR) as previously described [13]. Nitric oxide production by alveolar macrophages was assessed by measurement of supernatant nitrite using the Griess reaction (Parameter™ Total Nitric Oxide Assay Kit, R&D Systems; Minneapolis, MN).

### iNOS (NOS2) mRNA expression

Whole lung total RNA was isolated using the RNeasy Mini Kit (Qiagen). 10 ng of RNA was subjected to 2-step real-time reverse transcription and polymerase chain reaction (RT-PCR) using a pre-developed Taqman assay for mouse inducible nitric oxide synthase (iNOS or NOS2; Applied Biosystems, Foster City, CA) on the iCycler thermal cycler (Bio-Rad; Hercules, CA).

### Cytokine analysis

Cytokine levels in lung homogenate or cell culture supernatant samples were determined by Milliplex™ Map Kit bead array (Millipore; Billerica, MA) run on a BioPlex™ system (Bio-Rad).

### Histology

Lungs were fixed by inflation with 10% formalin to visual total capacity and embedded in paraffin. Lung sections were mounted onto slides and stained with hematoxylin and eosin and inspected by light microscopy at 40x and 100x magnification. The slides were evaluated by an experienced LSUHSC Morphology and Imaging Core microscopist without knowledge of treatment group assignment. Photomicrographs of representative sections were taken.

### Statistical analysis

Comparisons between different treatment groups were made using Student’s *t*-test for simple pair-wise comparisons. For data not normally distributed, values were log_10_ transformed prior to analysis. Survival functions were generated using Kaplan-Meier estimators, and comparisons between Kaplan-Meier survival curves were made using the log rank and Wilcoxon rank-sum tests. Differences between treatment groups were accepted as significant when *p* < 0.05.

## Results

### Bacteriology

**Figure 1:**
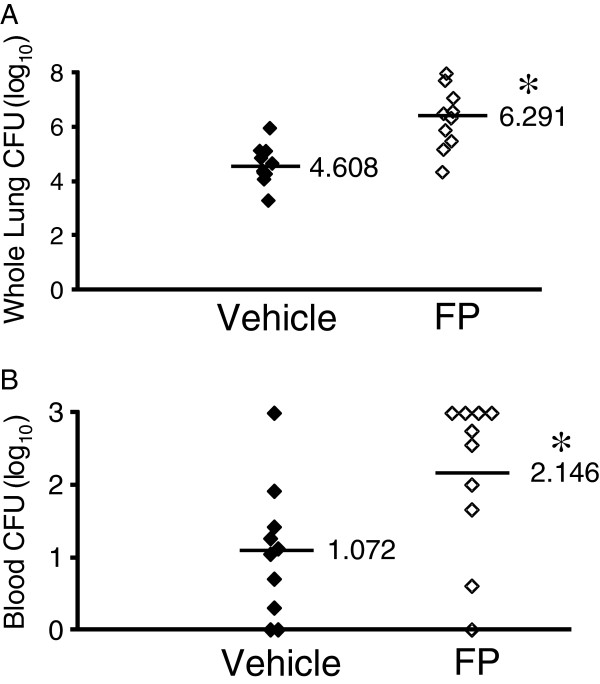
***K. pneumoniae*CFU in the lung (A) and blood (B) of FP or vehicle-treated mice 48 hours after i.t. challenge.** Mice receiving FP for 8 consecutive days prior to infection demonstrate significantly higher bacterial burdens in both compartments. n = 10 for each group. Lines indicate mean of each group. Results shown are from one of three separate experiments. * p < 0.05 vs. vehicle.

**Figure 2:**
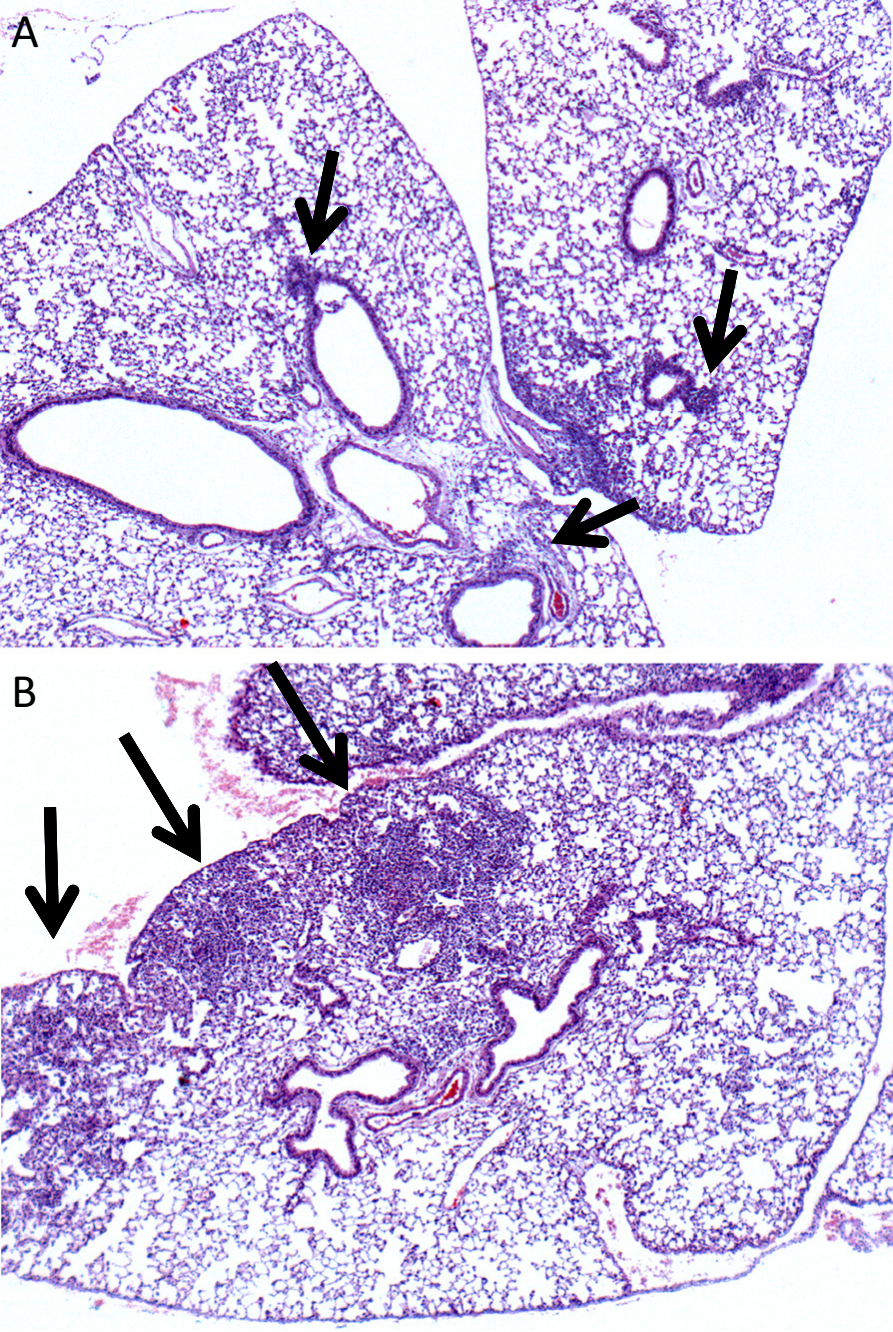
**Lung histology 24 hours after*K. pneumoniae*infection.** Lungs from control mice **(A)** show only patchy neutrophil infiltrates near the airways, whereas lungs from FP-treated mice **(B)** are already showing parenchymal consolidation with mild hemorrhage at this time point. Images are 40x magnification of H&E stained sections and are representative of findings from 4 mice in each group.

Our initial investigations sought to determine the effects of inhaled FP on the burden of bacteria present in the lungs and blood of mice at various intervals after an infectious i.t. dose of *K. pneumoniae*. CFU counts from the incubated whole-lung homogenates of FP-treated mice at both 4 hours and 24 hours after infection were higher than those from vehicle-treated animals, although the differences did not meet statistical significance (data not shown). However, experiments 48 hours after infection demonstrated a statistically significant increase in CFU counts from both the lungs and blood of FP-treated mice compared to vehicle-treated animals (Figure 1A and 1B). Histologic examination of lung tissues at 24 hours post-infection revealed only small areas of peri-bronchial neutrophil influx in control animals (Figure 2A), whereas lungs from FP-treated mice showed frequent and larger areas of confluent consolidation within the lung parenchyma (Figure 2B).

### Phagocytosis

Resident alveolar macrophages (AM) play a key role in the early innate response to infection in the lung. A notable function is their phagocytosis of free and opsonized bacteria in the alveolar spaces. Existing data suggest variable effects of different glucocorticoids on macrophage phagocytic function [14,15]. We conducted experiments to test the effects of inhaled FP on the in vivo phagocytosis of *K. pneumoniae* by mouse alveolar macrophages. The alveolar macrophages of mice exposed to FP, when compared to vehicle-exposed animals, demonstrated no difference in the percentage of macrophages which had engulfed bacteria by 2 hours after pulmonary bacterial challenge (51% vs 53%, control vs. FP, p = NS). The mean fluorescent intensity (MFI) of AM, indicating the number of bacteria per cell, consistently trended towards a greater amount in AM from FP-treated mice, although differences did not achieve statistical significance after adjustment for multiple comparisons (MFI of FP-treated AM 127% of control AM, p = 0.09).

### Cellular recruitment

**Figure 3:**
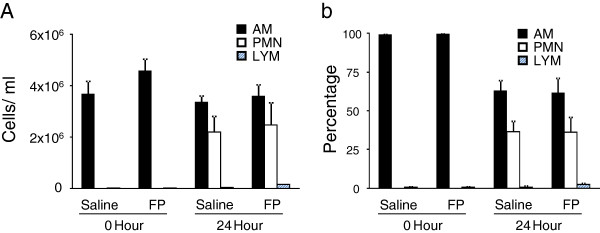
**BAL cell counts (A) and BAL cell differentials (B) of vehicle and FP-treated mice before and during*K. pneumoniae*challenge.** There were no observed differences between treatment groups in the total number of leukocytes recruited to the lungs or their differential composition before or 24 hours after bacterial challenge. n = 12 for each group (0 hour) and n = 18 for each group (24 hours).

Neutrophil recruitment is an essential component of the innate immune response to bacterial infection in the lungs. We investigated the effect of inhaled FP on neutrophil recruitment into the alveoli by performing BAL on the lungs of mice 24 hours after bacterial challenge. There were no significant differences in either the total number of leukocytes or the differential of leukocytes in BAL from mice nebulized with FP vs. vehicle (Figure 3A and 3B). BAL 16 hours post infection similarly showed no difference in early neutrophil recruitment (data not shown).

### Cytokines

**Figure 4:**
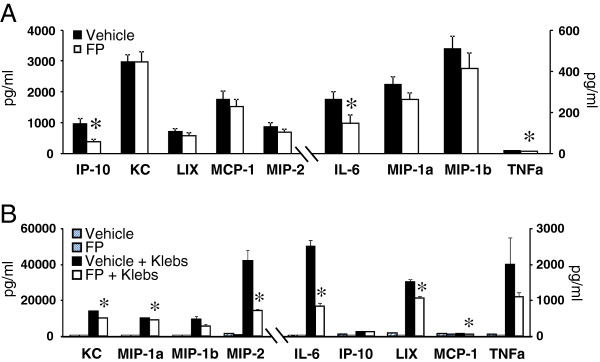
**A Cytokine expression in whole-lung homogenates 16 hours after i.t.*K. pneumoniae*challenge.** Shown are 9 cytokines of a 32-plex bead array analysis which were significantly induced by infection. Pretreatment with inhaled FP significantly attenuated the expression of IP-10, IL-6, and TNF-α 16 hours after infection. n = 6 per group. * p < 0.05 vs. vehicle. **B** Cytokine expression by murine AM before and 4 hours after in vitro stimulation with *K. pneumoniae*. While most cytokines measured showed up-regulation in response to *K. pneumoniae*, prior exposure to inhaled FP attenuated the expression of the majority of these. n = 6 per group. * p < 0.05 vs. vehicle + Klebs.

An extensive body of published research has demonstrated the importance of cytokine-mediated cell-cell interactions in both innate and adaptive immune responses to lung infection. Using a 32-plex cytokine bead array, we first analyzed whole-lung homogenates from FP and vehicle-treated mice 16 hours after *K. pneumoniae* infection and identified nine cytokines substantially up-regulated in response to bacterial challenge. Of these, IP-10, IL-6, and TNF-α showed significantly attenuated responses to infection in FP-exposed mice (Figure 4A). AM were then harvested from uninfected FP and vehicle-treated mice to assess their response to in vitro *K. pneumoniae* exposure. After four hours, culture supernatant was assayed for the same 9 cytokines. AM from FP-treated mice expressed significantly less KC, MIP-1α, MIP-2, IL-6, LIX, and MCP-1 compared to AM from vehicle-treated mice (Figure 4B). To confirm the effect of FP on AM cytokine release was both direct and dose-dependent, AM from uninfected mice (vehicle or FP-treated) were pre-treated (2 h) with FP (10 or 100 nM) followed by in vitro infection with *K. pneumoniae* (Table 1). Results for all 32 assayed cytokines are available as Additional file 1: Table S1.

**Table 1:** **Cytokine concentration (pg/ml) in cell culture supernatant following in vitro*K. pneumoniae*infection of Alveolar Macrophages**

**Vehicle-Exposed AM**	**IL-6**	**IP-10**	**KC**	**LIX**	**MCP-1**	**MIP-1a**	**MIP-1b**	**MIP-2**	**TNFa**
**PBS**	5.54 (4.54)	43.96 (2.44)	166.47 (11.12)	79.93 (15.99)	61.07 (5.78)	68.53 (4.30)	26.05 (8.24)	1,225.18 (36.94)	52.07 (0.93)
**Klebseilla**	2,506.37 (167.12)	122.53 (5.10)	13,917.04 (198.04)	1,518.32 (57.78)	68.74 (2.19)	10,038.19 (180.37)	9,318.00 (1,764.90)	41,802.27 (6,019.32)	2,012.61 (724.25)
**Klebs + 10 nm FP**	661.80* (36.50)	125.74 (3.51)	8,254.84* (268.01)	931.97* (21.59)	47.11* (1.71)	7,731.08* (161.64)	3,795.81* (200.74)	11,881.55* (622.15)	1,141.86 (100.01)
**Klebs + 100 nm FP**	472.29*# (23.18)	116.16 (6.96)	7,912.98* (271.24)	818.89* (22.82)	35.31*# (1.13)	6,674.62*# (252.86)	3,252.32*# (82.53)	11,441.20 (736.01)	1,101.10 (62.84)
**FP-Exposed AM**									
**PBS**	1.00 (0)	8.99 (1.23)	84.31 (1.72)	2.59 (1.59)	43.29 (5.49)	34.76 (2.98)	3.54 (2.54)	520.03 (26.90)	19.73 (0.85)
**Klebseilla**	828.87 (85.73)	122.38 (10.04)	9,891.14 (525.77)	1,056.19 (55.29)	50.38 (2.22)	9,111.51 (146.05)	5,640.23 (776.27)	14,296.83 (700.94)	1,096.82 (115.99)
**Klebs + 10 nm FP**	354.74* (23.31)	105.06 (5.47)	7,472.76* (459.38)	746.89* (45.01)	33.22* (1.35)	6,636.20* (378.28)	3,670.30* (228.33)	10,762.57* (557.97)	1,066.93 (71.74)
**Klebs + 100 nm FP**	327.28* (27.06)	107.73 (5.09)	6,492.42 (130.07)	701.09* (44.26)	25.64*# (0.90)	5,610.73*# (188.29)	3,511.53* (121.10)	8,612.62*# (355.80)	1,029.21 (122.45)

Cytokine expression by vehicle and FP-treated murine AM 4 h after in vitro stimulation with *K. pneumoniae.* The top four rows show mean and (SEM) cytokine responses by AM lavaged from vehicle treated mice, and bottom four rows are for AM from FP-treated mice. Some groups were also pre-treated in vitro for 2 h with FP (10 or 100 nM) before infection. Results suggest a dose-dependent effect of in vitro FP exposure on AM recovered from either naïve or FP nebulized mice. n = 6 per group. * p < 0.05 vs. *Klebsiella* exposure group; # p < 0.05 vs. *Klebsiella* + 10 nM FP group.

### Reactive oxygen/nitrogen species

**Figure 5:**
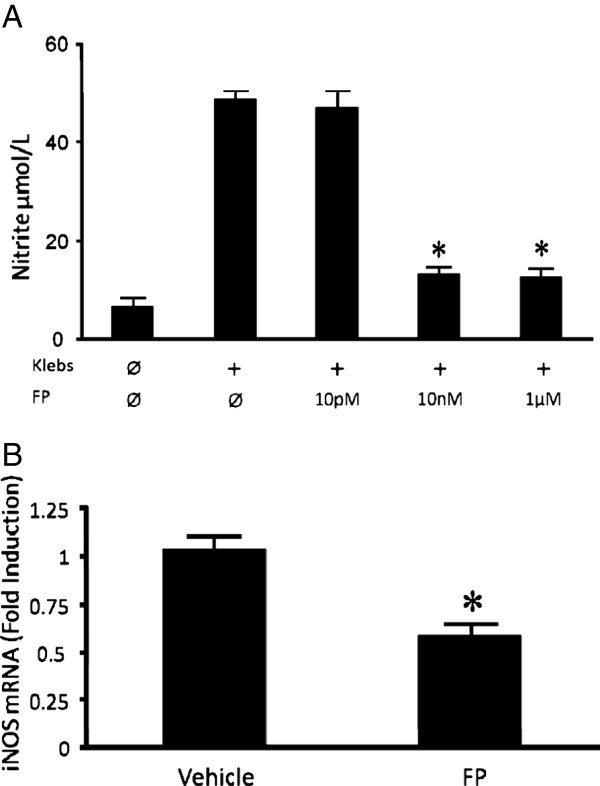
**A Overnight nitric oxide production by murine AM after*in vitro*stimulation with LPS (100 ng/ml) and rmIFN-γ (100 U/ml).** FP pre-treatment (2 h) decreased NO production at 10nM final concentration. n = 6 per group. * p < 0.05 vs. stimulated group without FP. **B** iNOS mRNA expression in the lungs of mice 90 minutes after i.t. *K. pneumoniae* challenge. Mice treated with FP for 8 days demonstrated significantly less iNOS mRNA after infection compared to vehicle-treated animals. n = 8 per group. * p < 0.05 vs. vehicle.

Once engulfed by phagocytic cells, bacteria are subjected to various bactericidal substances. Of such substances, reactive oxygen/nitrogen species (ROS/RNS) are among the most important. In light of significantly increased bacterial burdens in FP-treated mice in our pneumonia model, coupled with the lack of observed differences in phagocytic function conferred by FP, we conducted *in vivo* and *in vitro* experiments to investigate FP’s effect on AM expression of ROS/RNS. Initial experiments showed very little measureable H_2_O_2_ in the supernatant of *K. pneumoniae-*stimulated AM, whereas the phorbol ester PMA + ionomycin induced substantial extracellular release of H_2_O_2_*.* In vitro pre-treatment with FP at concentrations up to 1 μM had no effect on PMA-induced expression of H_2_O_2_ by AM harvested from either FP or vehicle-treated mice (1.32 vs. 1.49 uM H_2_O_2_ in FP vs. control AM respectively, p = NS). Extracellular release of nitric oxide by AM is also minimal in response to *K. pneumoniae.* FP pre-treatment did, however, reduce *K. pneumoniae-*induced AM nitric oxide expression in response to LPS/IFN-γ stimulation (Figure 5A). This in vitro finding was consistent with the demonstration of a significant decrease in the in vivo expression of iNOS mRNA in the lungs of FP-treated mice 90 minutes after bacterial infection (Figure 5B).

### Survival

**Figure 6:**
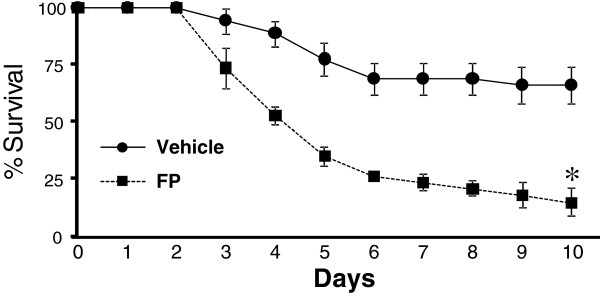
**Survival following i.t.*K. pneumoniae*challenge in FP and vehicle-treated mice.** Mice receiving FP for 8 consecutive days prior to infection demonstrate increased mortality compared to vehicle-exposed animals. Data shown are the cumulative results of three independent experiments. n = 34 for vehicle treatment and n = 36 for FP treatment. * p < 0.05 vs. vehicle.

Given the defects in bacterial clearance in the lungs and blood as a result of inhaled FP exposure, we sought to investigate the effect of inhaled FP on survival in this model of bacterial pneumonia. Our experiments demonstrate a significant increase in mortality in FP-treated mice compared to vehicle-treated mice after *K. pneumoniae* infection (Figure 6).

## Discussion

Here we show that a relatively brief (8 day) exposure to inhaled fluticasone propionate impairs pulmonary clearance of the gram negative pathogen *K. pneumoniae*, a finding associated with greater systemic bacterial burden and increased mortality compared to vehicle treatment. This effect was associated with decreased nitric oxide production and cytokine expression in the lungs. To our knowledge, this is the first work to explore the effects of inhaled corticosteroids on pulmonary host defenses against a virulent gram negative pathogen.

There is limited evidence detailing the effects of glucocorticoids on pulmonary host defense against bacterial pathogens. Adcock et al revealed a dose-dependent decrease in activation of the pro-inflammatory transcription factor NF-κB in lung epithelium treated with FP and then exposed to cytokine challenge [16]. Ek et al showed that treating human alveolar macrophages with FP impairs their release of cytokines in response to bacterial products [17]. We identified one in vivo study that demonstrated mice treated with inhaled FP exhibit decreased pulmonary neutrophil recruitment and decreased recovery of *M. pneumoniae* after inhalational inoculation [12]. While well-performed, these studies have limitations. For example, alterations in cytokine expression were measured in vitro and were in response to bacterial products rather than live bacteria*.* The in vivo murine ICS study characterized pulmonary immune responses to an atypical bacterium, one not identified as a cause of pneumonia in ICS-treated COPD patients [13]. Lastly, previous in vivo investigations of aerosolized corticosteroids used whole-body exposure models, which could result in significant systemic steroid delivery depending on animal grooming behavior and the oral bioavailability of the drug studied.

Despite the accumulated evidence demonstrating the therapeutic efficacy of ICS for treating obstructive lung disease, relatively few studies have examined the effects of these medications on pulmonary bacterial host defenses. Whereas studies have previously demonstrated measurable effects of *systemic* delivered corticosteroids on pulmonary responses to infectious agents such as *P. aeruginosaS. aureusL. pneumophilia*, and *L. monocytogenes*,[18-22] we found only a handful of studies detailing the effects of *inhaled* corticosteroids on the pulmonary host response. Chu et all found that FP impairs *M. pneumoniae* adhesion to epithelial cells [12]. Using a murine model of pneumonia similar to our current study, Barbier et al recently showed decreased airway epithelial cell adhesion by *Streptococcus pneumoniae and Haemophilus influenzae* in mice receiving a bolus dose of FP at time of infection [23]. By reducing epithelial cell expression of the platelet activating factor receptor (PAFR), FP decreased phosphorylcholine-mediated pathogen binding to PAFR. Interestingly, the pathogen in our current study, *K. pneumoniae*, does *not* express surface phosphorylcholine [24]. Given a recent analysis of the TORCH data demonstrating that *K. pneumoniae* was one of the four most commonly identified pneumonia pathogens in the COPD study population [11], we speculate that differences in pathogen characteristics may be critical in determining the effects of ICS on host susceptibility to bacterial pneumonia.

Alveolar macrophages play an important role in the initiation and orchestration of the acute response to lower respiratory tract infection, and they are responsible for the initial phagocytosis of deposited organisms. We were unable to demonstrate a defect in the *in vivo* capacity for alveolar macrophages to ingest fluorescently-labeled bacteria following FP treatment. These results contrast with earlier reports showing that in vitro phagocytosis of zymosan particles by sheep and rat alveolar macrophages is impaired by some glucocorticoids [14,15]. Key differences between prior studies and our current work include the in vivo study of phagocytosis, the use of live bacterial challenge, inhaled delivery of glucocorticoids, and the use of FP. Conversely, our flow data did show a trend (p = 0.09) towards more bacteria within AM lavaged from FP-treated mice, and we speculate this may represent a defect in early intracellular killing in vivo (as discussed separately). Nonetheless, we conclude that the initial phagocytosis of *K.* pneumoniae by AM’s is unaffected by our FP inhalation model.

Neutrophil influx is the histologic hallmark of acute bacterial pneumonia. Several studies have documented the importance of neutrophil recruitment in the host response to this infection [25,26]. Our results demonstrate increased lung parenchymal infiltration with neutrophils in FP-treated mice, likely a result of the increased burden of infection at this time point. Interestingly, we observed equivalent *alveolar* neutrophil recovery (by lavage) in FP and vehicle groups at this time point. These results are likely due a substantial percentage of the recruited neutrophils in the FP-mice being within the lung interstitium, such that recoverable (alveolar) neutrophil numbers are equivalent. At the least, these current results do not suggest a substantial quantitative defect in pulmonary neutrophil recruitment as a result of inhaled steroid treatment. Prior studies of steroids and pulmonary neutrophil recruitment are heterogeneous, employing various steroid agents, routes of administration, and inflammatory/infectious stimuli [12,22,27,28]. Given their diversity, it is not surprising that some studies show decreased pulmonary neutrophil recruitment while others do not. Our current work expands the knowledge of inhaled glucocorticoid effects on pulmonary innate immunity by demonstrating no clear modulation of neutrophil recruitment to live gram negative bacterial challenge, despite clear effects on lung bacterial clearance. This in vivo observation is particularly interesting in light of our in vitro data showing significant and dose-dependent inhibition of the neutrophil CXC chemokines MIP-2, LIX, and KC as secreted by AM 4 hours after bacterial challenge. However, the expression of the same chemokines in vivo was unaffected by FP exposure. We speculate that either 1) the differential burden of infection in vivo or 2) steroid-insensitive counter-regulatory mechanisms in vivo account for our finding of equivalent pulmonary neutrophil recruitment in FP-treated mice.

An important mechanism of bacterial killing is the host generation of reactive oxygen and nitrogen species [29,30]. Alveolar macrophage H_2_O_2_ production in response to PMA/I was unaffected by FP at doses up to 1 μM in our investigations, suggesting that this pathway is not steroid-sensitive. Alternatively, nitric oxide expression was inhibited by low doses of FP as demonstrated by our in vitro results. These findings are consistent with prior work showing decreased NOS2 induction by dexamethasone [21,31-33]. Our in vivo work extends these findings to live bacterial challenge by showing decreased iNOS mRNA in the lungs of FP-treated mice, and we speculate that the trend toward an increased number of bacteria found within AM from FP-treated mice may be related to their in vivo impairment of nitric oxide production during bacterial infection. While our in vitro data suggest that AM are an important cell population with decreased NO production, we speculate that numerous cell lines, including airway epithelial cells, may exhibit impaired NO expression as a result of ICS [34].

Our current study has important limitations. First, this is an animal study using a well-accepted model of pneumonia - one which causes mortality in a small but reproducible percentage of normal animals. By contrast, human trials show that the frequency of COPD exacerbations is diminished by ICS, and survival in the TORCH trial trended in favor of ICS treatment. Hence, we strongly caution against interpreting that these results can be extended to (or are representative of) ICS use in humans. Second, while our FP delivery was inhaled, we cannot exclude systemic FP effects in our model, particularly as a cause of increased bacterial burden in the bloodstream. While impaired lung bacterial clearance may be due to local FP effects, the inability to control systemic dissemination could also be attributable to extra-pulmonary effects of FP, since systemic effects of ICS are well-documented [35,36]. Lastly, while our data show defects in pulmonary cytokine responses and nitric oxide production, our in vitro studies are limited to AM and may not address other important cells, such as airway epithelium, in which host defense function is impaired.

## Conclusion

In summary, we demonstrate that a brief, 8-day exposure to inhaled FP interferes with normal murine innate pulmonary host defenses against the virulent gram negative pathogen *K. pneumoniae*, and this treatment leads to greater bacteremia and death. We believe that these results support the validity of clinical observations showing an increased risk of pneumonia in COPD patients treated with ICS. Given the widespread clinical use of ICS, further characterization of ICS effects on human pulmonary host defenses is warranted.

## Competing interests

KH has received consulting fees from GlaxoSmithKline. This project was funded by a collaborative research trial grant from GlaxoSmithKline to KH. No other authors have competing interests to disclose.

## Authors’ contributions

CP and RM performed the in vivo and in vitro experiments; AD performed the in vitro phagocytosis assays; XT performed the in vitro alveolar macrophage ROS/RNS experiments and assisted with the in vivo work. KH conceived the study, planned experimental design, and help draft the manuscript. All authors read and approved the final manuscript.

## Supplementary Material

Additional file 1 **Table S1.** Whole lung homogenate levels of cytokines 16 hours after *K. pneumoniae* infection. Data are expressed as pg/mg protein and are shown as mean (+/- SEM). FP = fluticasone propionate. * p < 0.05 vs. vehicle control. Click here for file

## References

[B1] National Asthma Education and Prevention Program Expert Panel Report 3: Guidelines for the Diagnosis and Management of Asthma 2007 NIH National Heart, Lung, and Blood Institute,

[B2] Rabe KF Hurd S Anzueto A Global strategy for the diagnosis, management, and prevention of chronic obstructive pulmonary disease: GOLD executive summary Am J Respir Crit Care Med 2007 176 6 532 555 10.1164/rccm.200703-456SO 17507545

[B3] Burge PS Calverley PM Jones PW Spencer S Anderson JA Maslen TK Randomised, double blind, placebo controlled study of fluticasone propionate in patients with moderate to severe chronic obstructive pulmonary disease: the ISOLDE trial BMJ 2000 320 7245 1297 1303 10.1136/bmj.320.7245.1297 10807619 PMC27372

[B4] Calverley P Pauwels R Vestbo J Combined salmeterol and fluticasone in the treatment of chronic obstructive pulmonary disease: a randomised controlled trial Lancet 2003 361 9356 449 456 10.1016/S0140-6736(03)12459-2 12583942

[B5] Mahler DA Wire P Horstman D Effectiveness of fluticasone propionate and salmeterol combination delivered via the Diskus device in the treatment of chronic obstructive pulmonary disease Am J Respir Crit Care Med 2002 166 8 1084 1091 10.1164/rccm.2112055 12379552

[B6] Calverley PM Anderson JA Celli B Salmeterol and fluticasone propionate and survival in chronic obstructive pulmonary disease N Engl J Med 2007 356 8 775 789 10.1056/NEJMoa063070 17314337

[B7] Ernst P Gonzalez AV Brassard P Suissa S Inhaled corticosteroid use in chronic obstructive pulmonary disease and the risk of hospitalization for pneumonia Am J Respir Crit Care Med 2007 176 2 162 166 10.1164/rccm.200611-1630OC 17400730

[B8] Ferguson GT Anzueto A Fei R Emmett A Knobil K Kalberg C Effect of fluticasone propionate/salmeterol (250/50 microg) or salmeterol (50 microg) on COPD exacerbations Respir Med 2008 102 8 1099 1108 10.1016/j.rmed.2008.04.019 18614347

[B9] Wedzicha JA Calverley PM Seemungal TA Hagan G Ansari Z Stockley RA The prevention of chronic obstructive pulmonary disease exacerbations by salmeterol/fluticasone propionate or tiotropium bromide Am J Respir Crit Care Med 2008 177 1 19 26 17916806 10.1164/rccm.200707-973OC

[B10] Singh S Amin AV Loke YK Long-term use of inhaled corticosteroids and the risk of pneumonia in chronic obstructive pulmonary disease: a meta-analysis Arch Intern Med 2009 169 3 219 229 10.1001/archinternmed.2008.550 19204211

[B11] Crim C Calverley PM Anderson JA Pneumonia risk in COPD patients receiving inhaled corticosteroids alone or in combination: TORCH study results Eur Respir J 2009 34 3 641 647 10.1183/09031936.00193908 19443528

[B12] Chu HW Campbell JA Rino JG Harbeck RJ Martin RJ Inhaled fluticasone propionate reduces concentration of Mycoplasma pneumoniae, inflammation, and bronchial hyperresponsiveness in lungs of mice J Infect Dis 2004 189 6 1119 1127 10.1086/382050 14999617

[B13] Zhou M Diwu Z Panchuk-Voloshina N Haugland RP A stable nonfluorescent derivative of resorufin for the fluorometric determination of trace hydrogen peroxide: applications in detecting the activity of phagocyte NADPH oxidase and other oxidases Anal Biochem 1997 253 2 162 168 10.1006/abio.1997.2391 9367498

[B14] Belayat F Meniai K Michaux C Kafidi N Coignoul F Dewaele A In vitro effect of glucocorticoids on phagocytic function of sheep alveolar macrophages Vet J 1998 155 2 177 181 10.1016/S1090-0233(98)80015-4 9564271

[B15] Nakamura Y Murai T Ogawa Y Effect of in vitro and in vivo administration of dexamethasone on rat macrophage functions: comparison between alveolar and peritoneal macrophages Eur Respir J 1996 9 2 301 306 10.1183/09031936.96.09020301 8777968

[B16] Adcock IM Nasuhara Y Stevens DA Barnes PJ Ligand-induced differentiation of glucocorticoid receptor (GR) trans-repression and transactivation: preferential targetting of NF-kappaB and lack of I-kappaB involvement Br J Pharmacol 1999 127 4 1003 1011 10.1038/sj.bjp.0702613 10433509 PMC1566089

[B17] Ek A Larsson K Siljerud S Palmberg L Fluticasone and budesonide inhibit cytokine release in human lung epithelial cells and alveolar macrophages Allergy 1999 54 7 691 699 10.1034/j.1398-9995.1999.00087.x 10442524

[B18] Blackwood LL Pennington JE Dose-dependent effect of glucocorticosteroids on pulmonary defenses in a steroid-resistant host Am Rev Respir Dis 1982 126 6 1045 1049 6983845 10.1164/arrd.1982.126.6.1045

[B19] Nash TW Libby DM Horwitz MA Interaction between the legionnaires’ disease bacterium (Legionella pneumophila) and human alveolar macrophages. Influence of antibody, lymphokines, and hydrocortisone J Clin Invest 1984 74 3 771 782 10.1172/JCI111493 6470140 PMC425231

[B20] Nugent KM Pesanti EL Chronic glucocorticosteroid therapy impairs staphylococcal clearance from murine lungs Infect Immun 1982 38 3 1033 1036 7152661 10.1128/iai.38.3.1033-1036.1982 PMC347853

[B21] Satoh S Oishi K Iwagaki A Dexamethasone impairs pulmonary defence against Pseudomonas aeruginosa through suppressing iNOS gene expression and peroxynitrite production in mice Clin Exp Immunol 2001 126 2 266 273 10.1046/j.1365-2249.2001.01656.x 11703370 PMC1906189

[B22] White JC Lanser ME Nelson S Jakab GJ Methylprednisolone impairs the bactericidal activity of alveolar macrophages J Surg Res 1985 39 1 46 52 10.1016/0022-4804(85)90160-X 4010275

[B23] Barbier M Agusti A Alberti S Fluticasone propionate reduces bacterial airway epithelial invasion Eur Respir J 2008 32 5 1283 1288 10.1183/09031936.00020608 18684852

[B24] Jansen HM Sachs AP van Alphen L Predisposing conditions to bacterial infections in chronic obstructive pulmonary disease Am J Respir Crit Care Med 1995 151 6 2073 2080 7767560 10.1164/ajrccm.151.6.7767560

[B25] Boe DM Nelson S Zhang P Quinton L Bagby GJ Alcohol-induced suppression of lung chemokine production and the host defense response to Streptococcus pneumoniae Alcohol Clin Exp Res 2003 27 11 1838 1845 10.1097/01.ALC.0000095634.82310.53 14634502

[B26] Ye P Rodriguez FH Kanaly S Requirement of interleukin 17 receptor signaling for lung CXC chemokine and granulocyte colony-stimulating factor expression, neutrophil recruitment, and host defense J Exp Med 2001 194 4 519 527 10.1084/jem.194.4.519 11514607 PMC2193502

[B27] Duong M Ouellet N Simard M Bergeron Y Olivier M Bergeron MG Kinetic study of host defense and inflammatory response to Aspergillus fumigatus in steroid-induced immunosuppressed mice J Infect Dis 1998 178 5 1472 1482 10.1086/314425 9780270

[B28] Rocksen D Lilliehook B Larsson R Johansson T Bucht A Differential anti-inflammatory and anti-oxidative effects of dexamethasone and N-acetylcysteine in endotoxin-induced lung inflammation Clin Exp Immunol 2000 122 2 249 256 10.1046/j.1365-2249.2000.01373.x 11091282 PMC1905762

[B29] Fang FC Antimicrobial reactive oxygen and nitrogen species: concepts and controversies Nat Rev Microbiol 2004 2 10 820 832 10.1038/nrmicro1004 15378046

[B30] Tsai WC Strieter RM Zisman DA Nitric oxide is required for effective innate immunity against Klebsiella pneumoniae Infect Immun 1997 65 5 1870 1875 9125574 10.1128/iai.65.5.1870-1875.1997 PMC175233

[B31] Skimming JW Nasiroglu O Huang CJ Dexamethasone suppresses iNOS yet induces GTPCH and CAT-2 mRNA expression in rat lungs Am J Physiol Lung Cell Mol Physiol 2003 285 2 L484 L491 12716655 10.1152/ajplung.00433.2002

[B32] Matsumura M Kakishita H Suzuki M Banba N Hattori Y Dexamethasone suppresses iNOS gene expression by inhibiting NF-kappaB in vascular smooth muscle cells Life Sci 2001 69 9 1067 1077 10.1016/S0024-3205(01)01196-1 11508649

[B33] Kunz D Walker G Eberhardt W Pfeilschifter J Molecular mechanisms of dexamethasone inhibition of nitric oxide synthase expression in interleukin 1 beta-stimulated mesangial cells: evidence for the involvement of transcriptional and posttranscriptional regulation Proc Natl Acad Sci U S A 1996 93 1 255 259 10.1073/pnas.93.1.255 8552616 PMC40217

[B34] Lehtimaki L Kankaanranta H Saarelainen S Turjanmaa V Moilanen E Inhaled fluticasone decreases bronchial but not alveolar nitric oxide output in asthma Eur Respir J 2001 18 4 635 639 10.1183/09031936.01.00000201 11716167

[B35] Chrousos GP Harris AG Hypothalamic-pituitary-adrenal axis suppression and inhaled corticosteroid therapy. 1. General principles Neuroimmunomodulation 1998 5 6 277 287 10.1159/000026348 9762010

[B36] Chrousos GP Harris AG Hypothalamic-pituitary-adrenal axis suppression and inhaled corticosteroid therapy. 2. Review of the literature Neuroimmunomodulation 1998 5 6 288 308 10.1159/000026349 9762011

