# Supplementary material for: Inhaled fluticasone propionate impairs pulmonary clearance of Klebsiella Pneumoniae in mice
Source: Respir Res. 2012 May 31;13(1):40. doi: 10.1186/1465-9921-13-40 (PMC3426464; doi:10.1186/1465-9921-13-40)
Supplement: Additional file 1 — Table S1. Whole lung homogenate levels of cytokines 16 hours after K. pneumoniae infection. Data are expressed as pg/mg protein and are shown as mean (+/- SEM). FP = fluticasone propionate. * p < 0.05 vs. vehicle control. [file 1465-9921-13-40-S1.doc]

**Supplemental Table 1**

|  | **Eotaxin** | **G-CSF** | **GM-CSF** | **IFNg** | **IL-1a** | **IL-1b** | **IL-2** | **IL-3** | **IL-4** |
| --- | --- | --- | --- | --- | --- | --- | --- | --- | --- |
| **Control** | 273.622  (14.212) | 665.257  (102.210) | 73.877  (7.747) | ND | 368.553  (45.482) | 162.178  (5.439) | 6.187  (0.483) | 2.748  (0.756) | 0.970  (0.112) |
| **FP** | 347.210  (25.826) | 481.737  (93.430) | 56.460  (9.141) | ND | 232.077  (25.305) | 140.855  (8.452) | 4.675  (0.233) | 1.540  (0.430) | 0.632  (0.098) |
|  | **IL-5** | **IL-6** | **IL-7** | **IL-9** | **IL-10** | **IL-12(p40)** | **IL-12(p70)** | **IL-13** | **IL-15** |
| **Control** | 28.737  (2.878) | 265.040  (34.616) | 8.575  (1.068) | 238.022  (15.932) | 18.025  (1.793) | ND | 26.870  (9.290) | 11.440  (0.00) | 9.498  (2.610) |
| **FP** | 13.670  (1.677) | 146.043*  (40.351) | 5.658  (1.127) | 147.315  (19.077) | 14.153*  (2.240) | ND | 24.958  (1.689) | 14.715  (7.637) | 5.263  (1.411) |
|  | **IL-17** | **IP-10** | **KC** | **LIF** | **LIX** | **MCP-1** | **M-CSF** | **MIG** | **MIP-1a** |
| **Control** | 24.740  (2.969) | 964.665  (158.796) | 2986.575  (206.900) | 3.582  (0.497) | 710.580  (94.564) | 1756.767  (268.061) | 34.585  (2.710) | 537.668  (130.429) | 337.285  (36.740) |
| **FP** | 14.565  (1.409) | 384.675  (62.895) | 2966.367  (341.009) | 2.658  (0.199) | 572.493  (92.240) | 1514.183  (235.958) | 27.057  (2.230) | 245.527  (77.584) | 261.930  (33.952) |
|  | **MIP-1b** | **MIP-2** | **RANTES** | **TNFa** | **VEGF** |  |  |  |  |
| **Control** | 511.368  (59.980) | 879.462  (115.047) | 150.997  (14.936) | 15.235  (1.658) | 113.642  (14.428) |  |  |  |  |
| **FP** | 413.700  (75.082) | 682.993  (91.878) | 81.880  (11.244) | 9.347*  (0.819) | 132.393  (32.169) |  |  |  |  |

Legend: Whole lung homogenate levels of cytokines 16 hours after *K. pneumoniae* infection. Data are expressed as pg/mg protein and are shown as mean (+/- SEM). FP = fluticasone propionate. * p < 0.05 vs. vehicle control.
